# See-saw-like movement of right heart

**DOI:** 10.1093/ehjcr/ytae572

**Published:** 2024-10-24

**Authors:** Fen Yu, Liming Gan, Yuqiang Shang, Jianxin Liu

**Affiliations:** Department of Ultrasound, The Central Hospital of Wuhan, Tongji Medical College, Huazhong University of Science and Technology, No. 26, Shengli Street, Jiangan District, Wuhan 430014, China; Department of Endocrinology, The Central Hospital of Wuhan, Tongji Medical College, Huazhong University of Science and Technology, No. 26, Shengli Street, Jiangan District, Wuhan 430014, China; Department of Cardiac Surgery, The Central Hospital of Wuhan, Tongji Medical College, Huazhong University of Science and Technology, No. 26, Shengli Street, Jiangan District, Wuhan 430014, China; Department of Ultrasound, The Central Hospital of Wuhan, Tongji Medical College, Huazhong University of Science and Technology, No. 26, Shengli Street, Jiangan District, Wuhan 430014, China

A 52-year-old female with no previous medical history was suspected of pericardial cyst on chest X-ray during routine examination, and electrocardiogram showed normal. Transthoracic echocardiography showed a large anechoic area pushing against the right heart, the free wall moved in a ‘see-saw’ motion, but the valves and the left heart were normal (*Figure 1A* and Supplementary material online, *Video S1*). Blood flow between the right atrium and the anechoic area remained undetectable until a meticulous sweep was conducted in the subcostal view. The width of the passageway measured 19 mm, and colour Doppler flow imaging disclosed systolic inflow and diastolic outflow (*Figure 1B* and Supplementary material online, *Video S2*). After injecting agitated saline into a superficial elbow vein, microbubbles entered a 91 × 57 × 74 mm cystic structure with thin walls and slow blood flow, which was consistent with right atrial appendage aneurysm (RAAA) (*Figure 1C* and Supplementary material online, *Video S3*). Computed tomography angiography confirmed a giant right atrial aneurysm without thrombosis (*Figure 1D*). Considering the risk of thromboembolism, the patient underwent aneurysmectomy. Intraoperative macroscopic and transoesophageal echocardiography showed that the aneurysm wall was thin and smooth without contraction (*Figure 1E* and *F*). Post-operative transthoracic echocardiography showed normal cardiac structure and wall motion.

**Figure 1:**
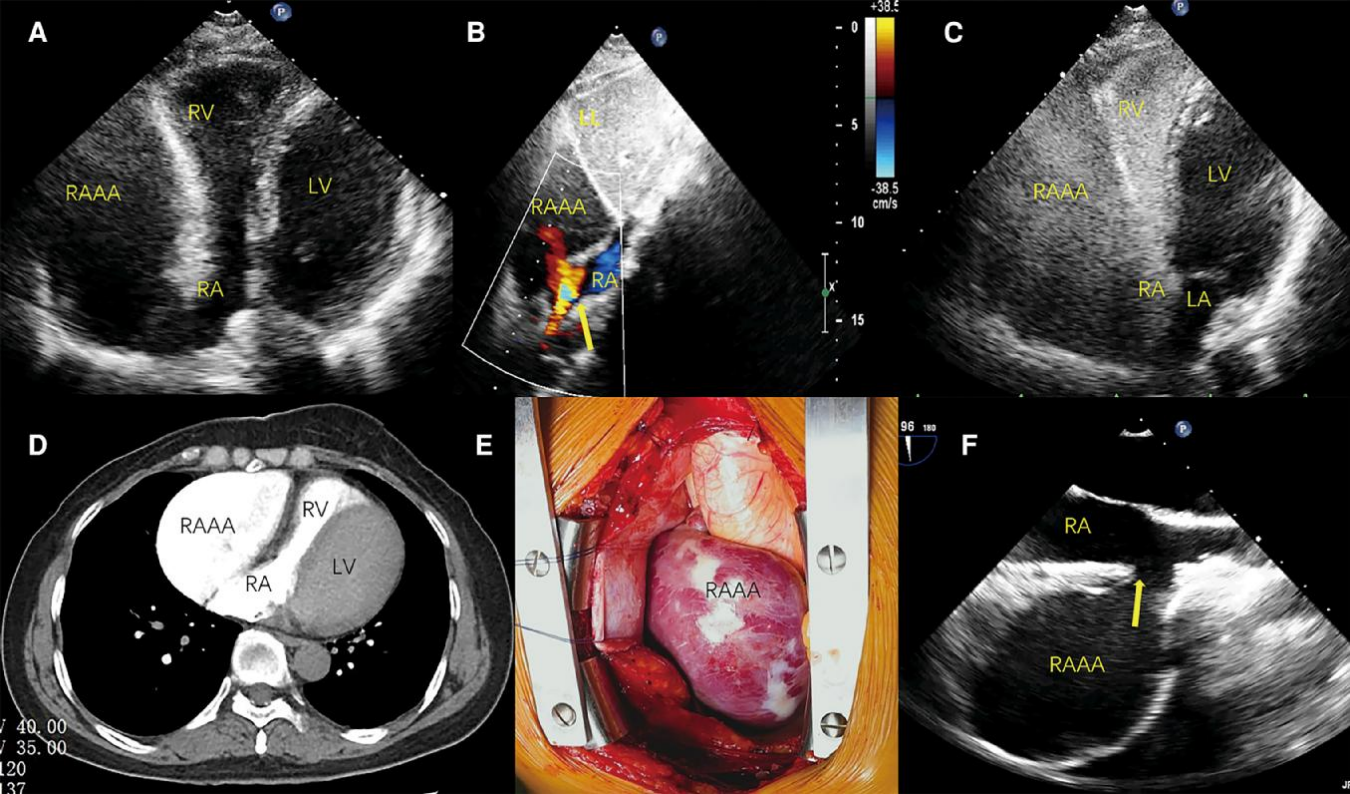
Right atrial appendage aneurysm (RAAA) on echocardiography. (*A*) The parasternal view shows RAAA pushing against the right heart. (*B*) In the subcostal view, the traffic between the RA and RAAA is observed (arrow). (*C*) The agitated saline microbubbles fill the right heart and RAAA. (*D*) Computed tomography angiography reveals no thrombus in the RAAA. (*E*) Intraoperative macroscopic shows the RAAA’s smooth surface. (*F*) Transoesophageal echocardiography displays RAAA’s smooth inner wall and the traffic between the RA and RAAA (arrow). RAAA, right atrial appendage aneurysm; RA, right atrial; RV, right ventricle; LV, left ventricle; LL, left lobe of liver; LA, left atrium.

Right atrial appendage aneurysm is a rare disease associated with a risk of thrombo-embolic complications or atrial arrhythmias, for which larger or enlarged RAAA is a predictive factor.^1^ Right atrial appendage aneurysm is usually diagnosed initially by transthoracic echocardiography and differentiated from pericardial effusion and other diseases.^2^ Agitated saline is helpful for differentiation. The giant RAAA located in the pericardium is affected by the relaxation and contraction of the heart and changes its size and shape, causing the right heart a see-saw-like movement, which is different from the ‘pendulum-like’ motion in the case of massive pericardial effusion and helpful for diagnosis.

## Supplementary Material

ytae572_Supplementary_Data

## Data Availability

All data analysed during this study are included in this article.

## Supplementary material

**Consent:** The authors confirm that written consent for submission and publication of this image and associated text has been received from the patient in line with COPE guidelines.

**Funding:** None declared.

## References

[1] Aryal MR, Hakim FA, Giri S, Ghimire S, Pandit A, Bhandari Y, et al Right atrial appendage aneurysm: a systematic review. Echocardiography 2014;31:534–539. 24447323 10.1111/echo.12510

[2] Wang Y, Liu A, Ye W. Congenital giant right atrial aneurysm: echocardiographic diagnosis and surgical management. Heart Surg Forum 2017;20:E055–E057. 28481744 10.1532/hsf.1656

